# Cooperative working of bacterial chromosome replication proteins generated by a reconstituted protein expression system

**DOI:** 10.1093/nar/gkt489

**Published:** 2013-06-03

**Authors:** Kei Fujiwara, Tsutomu Katayama, Shin-ichiro M. Nomura

**Affiliations:** ^1^Department of Bioengineering and Robotics, Tohoku University, 6-6-01, Aramakiaza-aoba, Aoba-ku, Sendai, Miyagi, 980-8579, Japan and ^2^Department of Molecular Biology, Graduate School of Pharmaceutical Sciences, Kyushu University, 3-1-1 Maidashi, Higashi-ku, Fukuoka, 812-8582, Japan

## Abstract

Replication of all living cells relies on the multirounds flow of the central dogma. Especially, expression of DNA replication proteins is a key step to circulate the processes of the central dogma. Here we achieved the entire sequential transcription–translation–replication process by autonomous expression of chromosomal DNA replication machineries from a reconstituted transcription–translation system (PURE system). We found that low temperature is essential to express a complex protein, DNA polymerase III, in a single tube using the PURE system. Addition of the 13 genes, encoding initiator, DNA helicase, helicase loader, RNA primase and DNA polymerase III to the PURE system gave rise to a DNA replication system by a coupling manner. An artificial genetic circuit demonstrated that the DNA produced as a result of the replication is able to provide genetic information for proteins, indicating the *in vitro* central dogma can sequentially undergo two rounds.

## INTRODUCTION

To answer the question ‘what is life?’ we must first learn to build a living cell. At least three distinct steps have been explored toward building cellular life: identification of the minimal genetic requirements by constructing a minimal cell (1,2), modification of living cells by introducing artificial genetic circuits (3) and reconstruction of systems in living cells from purified elements (4–7). Cell-free protein expression technology combines these three synthetic approaches. Artificial genetic circuits have been synthesized by cell-free protein expression (8,9), the reconstitution approach resulted in the creation of a minimum protein expression system by purified elements (PURE system) (6) and several biological subsystems have been reconstituted using the PURE system (10,11). However, these attempts have not been successful in reconstructing live cells from defined factors, and many problems remain to be solved, particularly the integration of these studies.

A simple method to integrate these synthetic studies is to reconstitute an *in vitro* system that consists of defined elements and that can progress through the central dogma processes multiple times (Supplementary Figure S1A). The PURE system contains >100 defined elements that are essential for transcription and translation in *Escherichia coli*, but lacks a DNA replication system (6). Thus, expression of chromosomal DNA replication system to the PURE system is a key approach to reconstitute *in vitro* central dogma cycles (Supplementary Figure S1B).

The process of *E. coli* chromosomal DNA replication occurs as follows (Supplementary Figure S2A) (12). DnaA molecules assemble on the origin of chromosomal replication (*oriC*), and separate the two DNA strands. DnaC loads DnaB helicase to the separated region. The loaded DnaB moves in a 5′ to 3′ direction while separating the duplex region, and recruits a primase (DnaG). DnaG synthesizes RNA primers for elongation with the DNA polymerase III holoenzyme (Pol III HE). In addition to these elements, several proteins, including DNA topoisomerase and DNA ligase, are also associated with the replication system. However, the five proteins described above are essential for replication of chromosomal-type DNA. On that point, the replication system for A-site single-stranded DNA (ssDNA; ABC primosome system) (13) is a good material to confirm the activity of proteins for chromosomal-type DNA replication. A-site ssDNA is a circular ssDNA with a hairpin-form duplex that has a DnaA binding site (Supplementary Figure S2B). Similar to chromosomal DNA, A-site ssDNA requires DnaA, DnaB, DnaC, DnaG and Pol III HE for replication. Successful replication of A-site ssDNA should indicate the existence of a chromosomal-type DNA replication system.

In this study, we reconstituted a central dogma cycle in *E. coli* by the production of a chromosomal-type DNA replication system using the PURE system (Supplementary Figure S1B). The *in vitro* central dogma cycle established here provides a fundamental framework toward reconstitution of multirounds central dogma system.

## MATERIALS AND METHODS

### *De novo* cell-free production of DNA replication proteins using the PURE system

**Figure 1.**
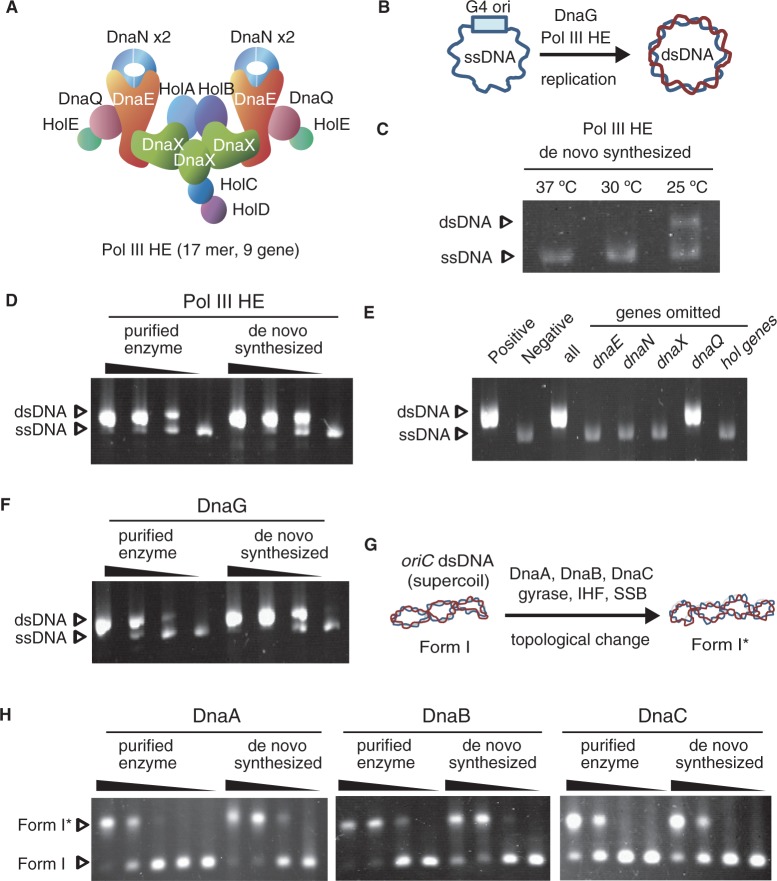
Functional DRPs are synthesized in PURE system. Proteins synthesized in the PURE system were used without purification for assays. SSB was firstly added to inhibit formation of heretoduplex due to T7 RNA polymerase transcriptional activity in the PURE system. (**A**) A representative figure of DNA Pol III HE. (**B**) A schematic representation of G4 replication assay. (**C**) Pol III HE expression at temperatures. The 9 genes that comprise Pol III HE were mixed with the PURE system at three temperatures, 37, 30 and 25°C. (**D** and **F**): *De novo* synthesized DnaG and Pol III HE could be replaced with purified enzymes. Pol III HE and DnaG were required for DNA replication of G4 ssDNA. Purified enzymes were used at 1.0-, 0.33-, 0.11- and 0-fold the amount indicated in the ‘Materials and Methods’ section. The *de novo* synthesized proteins were 3, 1 and 0.3 μl of the PURE system reaction mixture in 10 μl of total volume. As a negative control, 1 μl of the PURE system mixture without protein expression was used. Arrows indicated the corresponding bands of non-replicated DNA (ssDNA) and replicated dsDNA. (**E**) Essentiality of the 9 genes for Pol III HE expression was assessed by replication of G4 ssDNA. Positive: purified Pol III HE, Negative: no DNA polymerase, All: all of the 9 genes were synthesized using the PURE system. Genes omitted: each gene was omitted from the 9 gene mixture. The hol genes indicate the mixture of genes, holA, holB, holC, holD and holE. (**G**) A schematic representation of G4 replication assay. (**H**): Replacement assay of *de novo* synthesized DnaA, DnaB and DnaC with purified enzymes. Adding DnaA, DnaB, DnaC, SSB, IHF and gyrase alters the topology of DNA that have a chromosomal DNA replication origin (*oriC*). The topological differences were separated by low voltage gel electrophoresis. Purified enzymes were used at 1.0-, 0.33-, 0.11- and 0-fold of the amount indicated in ‘Materials and Methods’ section. The DnaA, DnaB and DnaC (DnaC was diluted into 1/16-fold with buffers) synthesized using the PURE system were used at 2.0, 0.67 and 0.22 μl in a 10 μl total volume. As a negative control, 2 μl of the PURE system mixture without protein expression was used. Arrows indicated the corresponding bands of form I and form I*.

The PURE system used in the present study is a commercial product, PUREfrex (GeneFrontier). The genes, *dnaB*, *dnaC*, *dnaG*, *dnaE*, *dnaN*, *dnaX*, *dnaQ*, *holA*, *holB*, *holC*, *holD*, *holE* and *ssb*, for DNA replication were amplified from chromosomal DNA of *E. coli* MG1655 strain. T7 promoter and SD sequence were attached to the genes except *dnaA* by second polymerase chain reaction (PCR), and to dnaA by cloning into the NdeI/XhoI site of pET29a (Merck). The negative control gene (*ftsZ*) was amplified from pWARA2 (14) by PCR. The amplified DNAs were purified using a PCR purification kit (Qiagen) or Wizard® SV Gel and PCR Clean-Up System (Promega). Total DNA concentrations were 1–3 nM as per the manufacture’s instructions. For Pol III HE expression, genes for *dnaE*, *dnaN*, *dnaX*, *dnaQ*, *holA*, *holB*, *holC*, *holD*, *holE* were mixed with 5:2:3:1:1:1:1:1:1:1 (ratio of weight). For G4 ssDNA replication in a single tube, the Pol III HE DNA mixture and *dnaG* was mixed 9:1 (ratio of weight). For replication of A-site ssDNA and T7GFP–A-site ssDNA in a single tube, *dnaA*, *dnaB*, *dnaC*, *dnaG* and the Pol III HE DNA mixture were mixed at 4.8:4.8:0.4:1.0:9.0 (ratio of weight). All DNA replication proteins (DRPs), except Pol III HE assay in Figure 1C, were synthesized using the PURE system for 15 h incubation at 27°C.

The purified DnaA were incubated with ATP for 10 min on ice to form the ATP complex just before use. Such preincubation was not required for the case of the DnaA synthesized by the PURE system because the PURE system contains ATP.

For coupling reactions, DNAs, 0.1 mM dNTP and single-strand binding protein (SSB) were added to the PURE system. SSB was mixed with ssDNAs before mixing with the PURE system. SSB coating of ssDNAs is required to avoid formation of heteroduplex due to T7 RNA polymerase transcriptional activity.

### G4 ssDNA replication assay

An ssDNA replication system from a G4 origin derived was performed as described previously (15). The mixtures were incubated for 5 min at 30°C in a 10 μl reaction volume containing G4 ssDNA (55 pmol as nucleotide), 20 mM Tris–HCl (pH 7.5), 8 mM dithiothreitol (DTT), 80 μg/ml bovine serum albumin, 4% sucrose, 10 mM magnesium acetate, 0.05 mM each of dNTP, 2 mM ATP, 0.2 mM each of GTP, CTP and UTP and 180 ng SSB. As controls, purified enzymes were used as follows: 18 ng DnaG and 53.6 ng Pol III HE.

### Form I* assay

Form I* assays were performed as described previously (16). Briefly, the indicated amounts of mixtures were incubated at 30°C for 30 min in buffer (10 μl) containing 20 mM Tris–HCl (pH 7.5), 0.1 mg/ml bovine serum albumin, 8 mM DTT, 10 mM magnesium acetate, 125 mM potassium glutamate, 2 mM ATP, 150 ng of SSB, 9 ng of IHF protein, 43 ng of ATP-DnaA, 21 ng of DnaB, 18 ng of DnaC, 90 ng of DNA gyrase A subunit, 98 ng of DNA gyrase B subunit and 40 ng of pBSoriC. In the case of replacement experiments with proteins produced by PURE system, the amounts of samples were indicated in Figure legends. The reaction was stopped in the presence of sodium dodecyl sulphate (SDS), EDTA and RNaseA.

### A-site ssDNA replication assay (ABC primosome assay)

ABC primosome assay was performed as described previously (16,17). Briefly, 10 μl solution that contains A-site ssDNA (90 pmol as nucleotide), 20 mM Tris–HCl (pH 7.5), 0.1 mg/ml bovine serum albumin, 8 mM DTT, 8 mM magnesium acetate, 0.01% Brij-58, 125 mM potassium glutamate, 1 mM ATP, 0.25 mM each of GTP, CTP and UTP, and 0.1 mM each of dNTP, and 0.3 μg of SSB were used. As positive controls, purified enzymes were used as follows: 43 ng of DnaA-ATP, 26 ng of DnaB, 26 ng of DnaC, 18 ng of DnaG, 53.6 ng of Pol III HE. In the case of replacement experiments with proteins produced by PURE system, the amounts of samples were indicated in Figure legends. The mixtures were incubated at 30°C for 15 min.

### Plaque-forming assay for biological activity of the replicated DNA

The replicated DNA was purified by RNase A, SDS, phenol/CHCl_3_ and ethanol precipitation. Ten microliters *E. coli* JM109 F(+) competent cells were transformed using 1 μl of the purified DNAs, and the same strain grown in LB medium was used as recipient cells in top agar. The total numbers of plaque formed were counted.

### Artificial genetic circuit assays to detect two-round flow of the central dogma system

T7GFP–A-site ssDNA was constructed as follows. A-site replication origin was attached to the superfolder green fluorescent protein (GFP) (18) under T7 promoter (Figure 4A) by PCR, and cloned into the PstI/EcoRI site of M13mp18RF (TAKARA). The T7GFP–A-site ssDNA in the resultant M13 phage was purified with the QIAfilter Plasmid Maxi Kit (Qiagen). Mixtures of the T7GFP–A-site ssDNA (180 pmol as nucleotide), the 13 genes, 0.1 mM dNTP and SSB were added to the PURE system. After a 15-h incubation at 27°C, half of the samples after treatment with RNaseA, phenol/CHCl_3_ and ethanol precipitation, were electrophoresed in agarose gels, and stained with SYBR Gold gel stain (Invitrogen). The remaining samples (half) without boiling were electrophoresed by SDS-polyacrylamide gel electrophoresis to detect GFP expression.

### Electrophoresis

All DNA samples were electrophoresed by 0.65% agarose gel in 0.5 × Tris/Borate/EDTA (TBE) for 1 h at 50 V for the replication assays, and 16 h at 20 V for the Form I* assay. Gels were stained with ethidium bromide except the assay for two-round flow of the central dogma system. The migration position of ssDNA and double-stranded DNA (dsDNA) molecules were determined by untreated ssDNA and replicative form of dsDNA made by purified proteins in all experiments.

## RESULTS

### Reconstitution of Pol III HE complex using a reconstituted protein expression system

Pol III HE is a hetero protein complex that is composed of 17 subunits and 9 different proteins (Figure 1A) (19–21). Because of the complexity, first, we attempted to identify the proper conditions to express the functional Pol III HE complex. According to comprehensive proteomics data from Niwa *et al.*, many Pol III HE components are inherently aggregation prone (Supplementary Figure S3) (22,23). However, *E. coli* lacking two major molecular chaperones can grow at temperatures of <30°C, and DRPs have not been identified as obligate substrates of the only known *E. coli* chaperone required for growth (24,25). These pieces of evidence suggest that lower temperatures are ideal candidate conditions for producing functional Pol III HE without the presence of molecular chaperones. To test this, we expressed Pol III HE using the PURE system at three temperatures: 37, 30 and 25°C. Pol III HE activity was measured by a G4 phage ssDNA replication assay, which only requires primase (DnaG) and Pol III HE for replication (Figure 1B) (15). Because the absence of the SSB induced the unexpected formation of duplex bands by a PURE system component (Supplementary Figure S4AB), SSB was mixed with DNA before adding the PURE system. Without SSB, the only polymerase in the PURE system, T7 RNA polymerase, caused heteroduplex formation of ssDNA (Supplementary Figure S4C) (see Discussion). As expected, Pol III HE synthesized in the PURE system at 25°C showed DNA replication activity, but not at temperatures >30°C in the presence of purified DnaG (Figure 1C). Consequently, overnight expression (∼15 h) at 27°C using a fresh PURE system produced functional Pol III HE at levels comparable with purified Pol III HE (Figure 1D). Under this condition, the 13 proteins required for A-site ssDNA replication were successfully expressed by PURE system (Supplementary Figure S5). Omitting any of the DNA of components of Pol III HE (*dnaE*, *dnaN*, *dnaX* and *hol* genes) from the mixture diminished the DNA replication activity. However, omitting *dnaQ* DNA, which encodes a proofreading exonuclease, did not affect the replication activity (Figure 1E). These results are consistent with previously published data [total reconstitution of Pol III HE by purified enzymes (19), and identifying the essential *E. coli* genes required for growth (26)]. These evidences indicated successful reconstitution of Pol III HE complex, and raised the first direct evidence of that polypetpides chains emerged from ribosomes spontaneously assembled into Pol III HE.

### RNA priming and DNA replication by *de novo* synthesized factors through PURE system

Second, primase was synthesized by using PURE system. DnaG activity was examined by replication of G4 ssDNA using DnaG synthesized in the PURE system and purified Pol III HE. Replication of G4 ssDNA is initiated by binding of DnaG on the G4 origin and primer synthesis. Overnight expression at 27°C of the PURE system resulted in synthesis of functional DnaG primase (Figure 1F). Mixing the cell-free synthesized Pol III HE and DnaG resulted in successful replication of G4 ssDNA in the absence of the purified enzymes (Supplementary Figure S6). In this way, we found the PURE system is able to produce active systems for RNA priming and elongation of DNA replication.

### Initiation machineries synthesized in PURE system without chaperones

Third, we performed a Form I* assay to test for the production of initiation machineries by the PURE system. Mixtures of DnaA, DnaB, DnaC, gyrase, IHF and SSB cause specific duplex unwinding of supercoiled (Form I) *oriC* DNA and subsequent expansion of the ss region, resulting in changes in the linking number of supercoiled *oriC* DNA by gyrase (27). The resultant topoisomers of Form I DNA are called Form I*. Form I* can be distinguished from Form I by different migration rate in gel electrophoresis. The Form I* assay can be thus used to examine DnaA initiator activity, and DnaB helicase and DnaC helicase-loader activities in replication initiation at *oriC* (Figure 1G). These three proteins were also synthesized in the PURE system at 27°C overnight. Replacement of each of the purified proteins in the Form I* assay revealed that the cell-free synthesized proteins were essentially active (Figure 1H). Although previous studies suggested molecular chaperones contribute some process of initiation (28,29), our results showed the proteins examined here fold spontaneously into their correct structures at least under 27°C. Taken together, we revealed that the PURE system is able to produce all the components necessary for A-site ssDNA replication without molecular chaperones.

### Replication of a model DNA system by mixtures of the *de novo* synthesized proteins in PURE system

**Figure 2.**
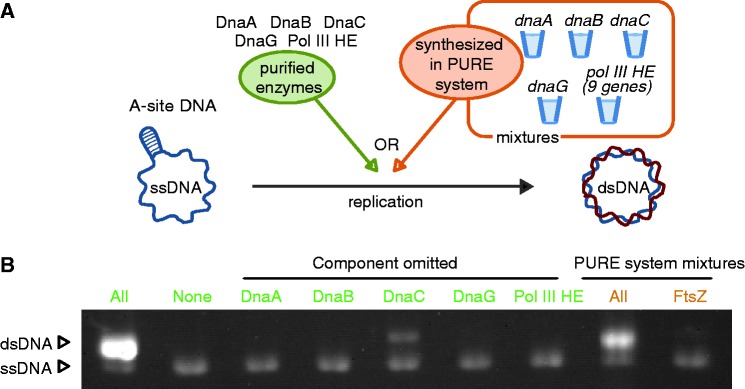
A mixture of DRPs that are expressed by the PURE system works in concert to replicate chromosomal DNA. (**A**) A schematic representation of A-site ssDNA replication assay. (**B**) DRPs were synthesized by using the PURE system in different tubes, and then mixed to examine A-site ssDNA replication. Green characters indicate purified proteins were used, and magenta characters indicate that *de novo* proteins synthesized through the PURE system were used. All: DnaA, DnaB, DnaC, DnaG and Pol III HE were used for DNA replication. None: no DRPs were added. Component omitted: each protein described above lanes was not added the reaction mixtures. All (PURE system mixtures): 1.6, 1.5, 0.12, 1.5 and 3.0 μl of DnaA, DnaB, DnaC, DnaG, Pol III HE synthesized in PURE system were mixed in the 10-μL final mixture. FtsZ: PURE system expressing *ftsZ* gene was used as a negative control because solution from PURE system mixtures comprises 80% of the volume of total mixtures. SSB was firstly added to inhibit formation of heretoduplex due to T7 RNA polymerase transcriptional activity in the PURE system.

Individual functionality of DRPs does not suggest that these factors are able to work as a system. Thus forth, to demonstrate that the DRPs synthesized by using PURE system work as a system, we assessed whether mixtures of these synthesized proteins replicate A-site ssDNA (Figure 2A). Under our conditions, omitting purified enzymes DnaA, DnaB, DnaG and Pol III HE did not result in replication of A-site ssDNA, and omitting DnaC exhibited slight replication (Figure 2B). The mixtures of the DRPs that were expressed by the PURE system in different tubes showed efficient replication of A-site ssDNA (Figure 2B). The replication levels were slightly lower than when purified enzymes are used, but much higher than the negative controls. The PURE system containing the *ftsZ* gene (a bacterial tubulin homolog) as a negative control did not show replication activity. These results indicate that proteins synthesized in the PURE system can work as a system for DNA replication.

### A chromosomal DNA replication system spawned in a single PURE system tube

**Figure 3.**
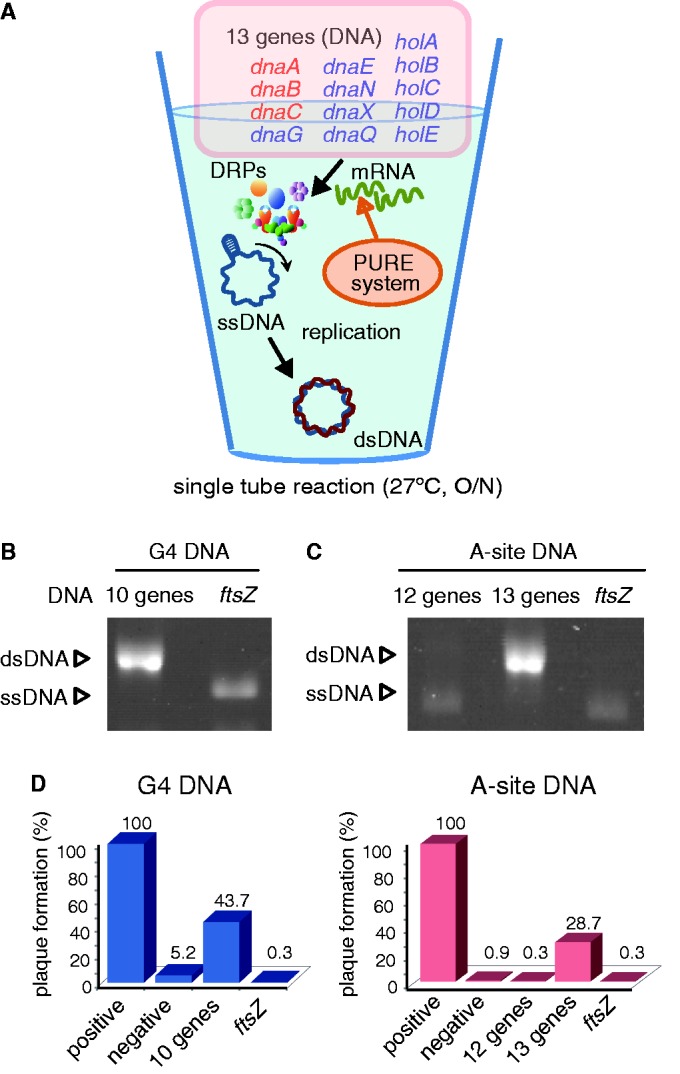
Autonomous DNA replication by expression of DRPs using PURE system in a single tube. (**A**) A schematic diagram of a coupling reaction in which protein expression induces DNA replication. The 10 genes for G4 ssDNA replication and the additional 3 genes for A-site ssDNA replication are indicated by blue and red characters, respectively. (**B**, **C**): The result of the coupling reaction, in which PURE system synthesizes DRPs, and DRPs replicated DNA in a single tube. (B, C): SSB was added before DRPs synthesis by the PURE system. (B) ‘negative’, ‘10 genes’ and ‘*ftsZ*’ indicate no DNA for protein expression, the 10 genes described by blue character in Fig 3A, and *ftsZ* gene as a negative control were added, respectively. (C) ‘negative’, ‘13 genes’ and ‘*ftsZ*’ indicate no DNA, the 13 genes described in Fig 3A and *ftsZ* gene as a negative control were added, respectively. In the case of the 12 genes, *dnaC* gene was omitted from the 13 genes. (**D**): Plaque-forming assay for evaluating biological activity of the replicated DNA by the coupling reaction in a single tube. DNA purified through phenol/CHCl_3_ and RNaseA treatment was used. Relative units indicate the rate of plaque numbers, when the plaque number of the replicated DNA by purified enzymes was determined as 100%. The 100% indicates 615 plaques for G4 ssDNA and 1434 plaques for A-site ssDNA.

Fifth, we reconstituted the cycle of central dogma in a single tube (Figure 3A). The 10 genes (*dnaG* and 9 genes for Pol III HE) for G4 ssDNA or the 13 genes for A-site ssDNA were mixed with the PURE system. After overnight incubation at 27°C, the full set (the 10 genes for G4 ssDNA or the 13 genes for A-site ssDNA) showed DNA replication coupled with transcription and translation in a single test tube (Figure 3B and C). Fluorescent-tagged dUTP showed dNTP were actually incorporated into the newly synthesized DNA (Supplementary Figure S7). The relative replication efficiencies estimated from intensities of the bands were 108% for G4 ssDNA and 103% for A-site ssDNA when compared with those by purified DRPs. The results suggest that the DNA replication by the *in vitro* coupled reaction proceeded only once. In contrast, the *ftsZ* gene negative control or omitting the *dnaC* gene (12 genes) did not result in obvious DNA replication.

By using the ssDNAs, we performed a phage plaque-forming assay to confirm the biological activity of the DNA replicated in the *in vitro* coupling reaction (Figure 3D). The G4 ssDNA and A-site ssDNA are modified bacteriophage f1/M13 DNA, and have ability to form infectious phages from their DNA sequences (13,15). A life cycle of ssDNA phages is described as follows. Firstly, phages attach to *E. coli* cells to transfer their ssDNA into cells. The complementary strand of transferred ssDNA is synthesized by DRPs of *E. coli*. The synthesized dsDNAs of phage genome are amplified through rolling circle mechanism by *E. coli* and phage proteins, and then are coated by phage capsids. Resultant phages are released through cell lysis and infect other *E. coli* cells again, which makes plaques on solid media. Thus, if phage DNA were transferred into cells, plaques were formed through the DNA. Because transformation efficiency of cells with ssDNA is lower than that with dsDNA, plaque forming shows biological activity of the replicated dsDNA. Thus we performed this assay after G4 ssDNA, and A-site ssDNA were replicated in the coupled system (Figure 3D). The resultant DNA showed significant plaque formation, although the numbers of plaques were 2.2- (G4 ssDNA) or 3.5- (A-site ssDNA) fold lower than that of DNA replicated by purified enzymes. The DNA used for protein expression in the PURE system might be the cause of the plaque formation decrease because the number of plaques formed when only the ftsZ gene is added was less than the negative control. Nevertheless, it is evident that a reconstituted protein expression system, the PURE system, exhibits DNA replication in a single test tube. These results showed PURE system are able to process sequential transcription–translation–replication in a single tube.

### Two-rounds flow of the reconstituted *in vitro* central dogma revealed by an artificial gene circuit

**Figure 4.**
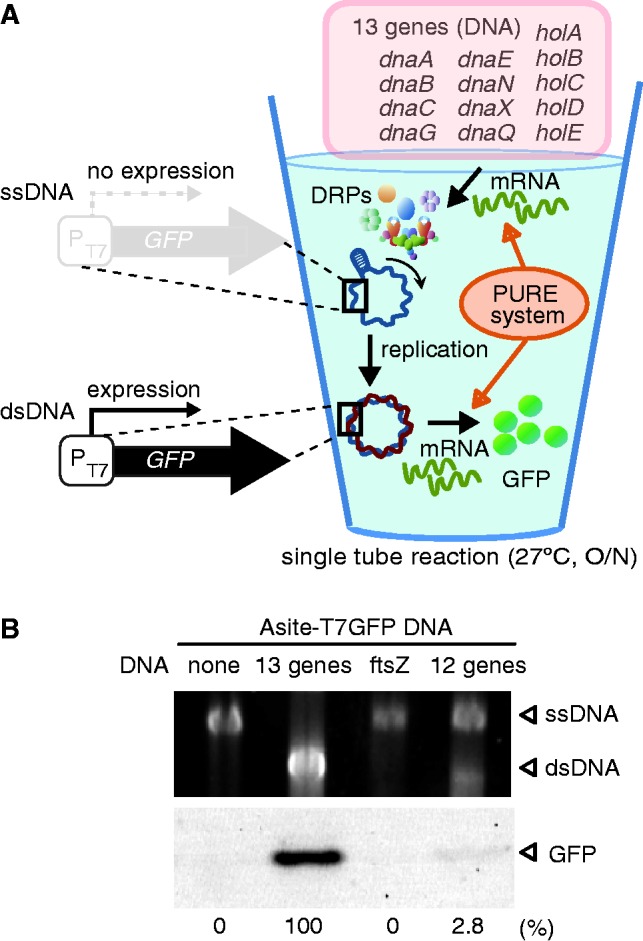
Two-round flow of the reconstituted central dogma system revealed by an artificial genetic circuit. (**A**) Schematic representation of the artificial genetic circuit to detect two-round flow of the reconstituted central dogma. An ssDNA that has T7-GFP sequence and A-site origin (T7GFP–A-site ssDNA) was used. Only the replicated form of T7GFP–A-site DNA is able to express GFP. In the artificial circuit, the PURE system with the 13 gene synthesizes DRPs. DRPs replicated T7GFP–A-site ssDNA, and the replicated DNA provides information for GFP expression. Thus, GFP expression indicates the reconstituted central dogma works two rounds. (**B**) Experimental results of two-round central dogma assay in a single tube. SSB was added before DRPs synthesis by PURE system. The upper panel shows DNA replication of T7GFP–A-site ssDNA. In the case of T7GFP–A-site ssDNA under our conditions, migration of the replicated DNA is faster than the not-replicated DNA. Arrows indicated the corresponding bands of the not-replicated DNA (ssDNA) and of the replicated dsDNA. The lower panel shows GFP expression through the artificial genetic circuit. The ‘12 genes’ indicates omission of the *dnaC* gene from the 13 genes. All samples used in this experiment contain T7GFP–A-site ssDNA. The case of the ‘13 genes’, 127 nM of GFP was expressed.

Finally, we constructed an artificial circuit that can detect two central dogma rounds of flow. In this construct, the *gfp* gene is regulated by the T7 promoter, which is located upstream of the A-site origin of replication in an inverse orientation, and thus the T7 promoter is active only after replication (Figure 4A). The ss form of T7GFP–A-site DNA was migrated slower than the ds form (Supplementary Figure S8). It has been known that ssDNA is more sensitive to electrophoresis conditions than ds DNA. Because the migration point of the ds form of T7GFP–A-site DNA located around the predicted position (∼8 kb), the slower migration of the ss form might be caused by electrophoresis condition we used. The 13 genes (*dnaA*, *dnaB*, *dnaC*, *dnaG*, *dnaE*, *dnaN*, *dnaX*, *dnaQ*, *holA*, *holB*, *holC*, *holD*, *holE*) were cell-free translated by the PURE system using the artificial circuit. Similar to the case of the A-site ssDNA, the 13 genes resulted in efficient DNA replication and GFP expression, while negative controls (expressing the *ftsZ* gene or without the genes) did not (Figure 4B). Omitting *dnaC* (12 genes) drastically decreased GFP expression and DNA replication. These data indicate that DNA was replicated via the products of the 13 genes in the PURE system, and that GFP was expressed as a result of the sequence information provided by this replicated DNA. Hence, we concluded the *in vitro* central dogma works two rounds in a single tube.

## DISCUSSION

Reconstitution of biological systems by the PURE system contributes to realize *in vitro* self-replication system. Subsystem of lipids synthesis from 2 genes (10), RNA polymerase and sigma factor cascade system from 5 genes (11) have been reconstituted by the PURE system. However, we should note that only 30–50% of total proteins in *E. coli* form soluble structure when expressed by the PURE system under chaperone-free condition (22,23). In addition, yields of proteins by the PURE system are another limitation of the reconstitution of a biological system. Thus, greater number of components the system contains, it becomes more difficult to achieve reconstitution of the target system by the PURE system. In this context, this study is a remarkable achievement because it found adequate conditions so that the PURE system can reconstitute a multicomponent system, chromosome type DNA replication, using 13 genes.

Our research also has several new findings in the PURE system and DRPs. Formation of heteroduplex from ssDNA by T7RNA polymerase in the absence of SSB (Supplementary Figure S4) has not been reported. This could depend on specific conditions of the PURE system or for formation of the secondary structure of ssDNA, which can activate T7RNA polymerase. Furthermore, we showed that temperature <30°C is a necessary condition for producing functional Pol III HE by the PURE system (Figure 1C), although *E. coli* cells can survive above this temperature. The difference may be originated from lack of chaperones in the PURE system. Previous studies by genetics (24), proteomics (25) and comprehensive protein expression (22,23) suggested that chaperones are indispensable for production of genomic DNA replication machineries at >30°C. Our results strongly supported the notion, and connected studies on protein-folding and DNA-replication system.

Recent studies have reported the synthesis of the whole bacterial genome and the creation of an artificial bacterial strain by genome transplantation (30,31). Furthermore, recently, highly condensed functional cell extracts that have similar levels of macromolecules as living cells were generated (32), and bacteriophages T7 were synthesized using cell extract (33). These achievements inspire hope to reconstitute live cell from defined factors in near future. However, challenges still remain even in *in vitro* DNA replication system. For example, reconstitution of DNA replication cycles *in vitro* (12) and achievement of *in vitro* replication of long chromosomal DNA over 4 Mb are the matter. An easy procedure to identify substances that may lack in the present *in vitro* reconstituted DNA replication system is dispensable for overcoming the challenges.

As shown in this study, a defined protein expression system with a set of genes is able to generate a biological system that proceeds as a multistep process like DNA replication. Especially, the procedure for reconstitution of Pol III HE in this study is much simpler than a simplified method reported recently (34). Possibility of the generated system will be expanded by only adding further genes. All genes in *E. coli* have been individually cloned into plasmids (35). Expanding the present study should illuminate unrevealed substances toward *in vitro* reconstitution of multirounds central dogma system.

## SUPPLEMENTARY DATA

Supplementary Data are available at NAR Online: Supplementary Figures 1–8 and Supplementary References [36–38].

## FUNDING

Funding for open access charge: Japan Society for the Promotion of Science, KAKENHI [22220001, 24104004, 23.3718].

*Conflict of interest statement*. None declared.

## Supplementary Material

Supplementary Data
