# Supplementary material for: Cooperative working of bacterial chromosome replication proteins generated by a reconstituted protein expression system
Source: Nucleic Acids Res. 2013 Jun 3;41(14):7176–83. doi: 10.1093/nar/gkt489 (PMC3737561; doi:10.1093/nar/gkt489)
Supplement: Supplementary Data [file supp_41_14_7176__index.html]

Cooperative working of bacterial chromosome replication proteins generated by a reconstituted protein expression system — Cooperative working of bacterial chromosome replication proteins generated by a reconstituted protein expression system — Supplementary Data 

# Cooperative working of bacterial chromosome replication proteins generated by a reconstituted protein expression system

## Supplementary Data

files

**Files in this Data Supplement:**

- Supplementary Data - pdf file
