# Supplementary material for: Cooperative working of bacterial chromosome replication proteins generated by a reconstituted protein expression system
Source: Nucleic Acids Res. 2013 Jun 3;41(14):7176–83. doi: 10.1093/nar/gkt489 (PMC3737561; doi:10.1093/nar/gkt489)
Supplement: Supplementary Data [file supp_gkt489_nar-00897-v-2013-File006.pdf]

Cooperative working of bacterial chromosome replication proteins generated by a reconstituted protein expression system

Kei Fujiwara*, Tsutomu Katayama†, and Shin-ichiro M. Nomura*

* Department of Bioengineering and Robotics, Tohoku University, Japan

† Department of Molecular Biology, Kyushu University, Japan

Supplementary Figures legends

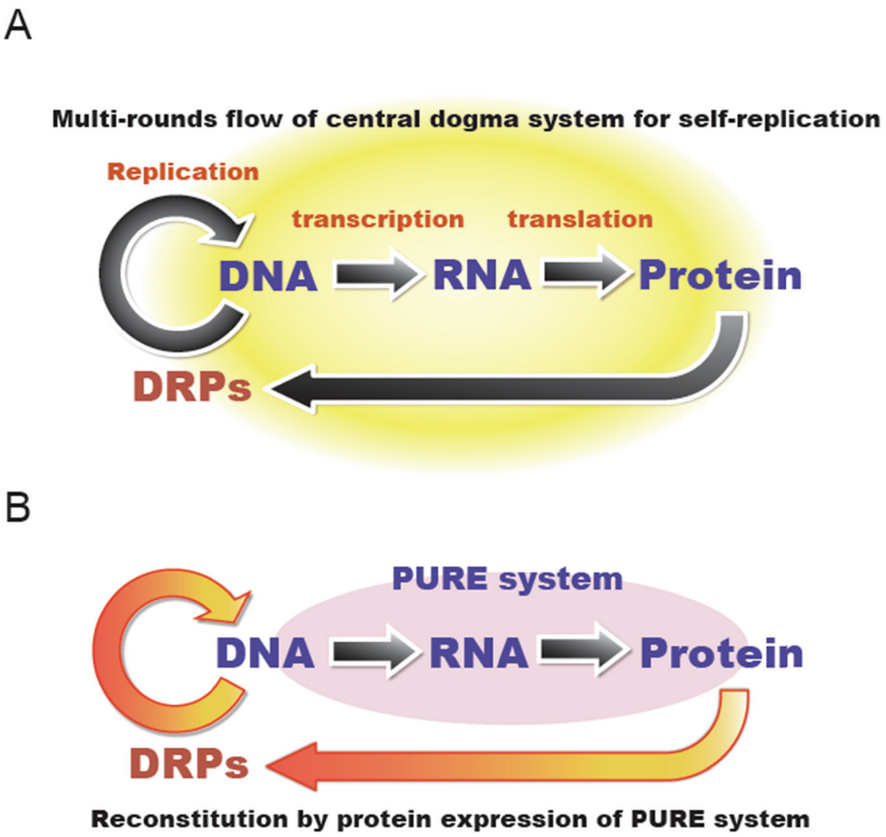

Figure S1. Reconstitution of multi-rounds of flow of the central dogma system

A: Multi-rounds of flow of the central dogma system for self-replication. The original central dogma of molecular biology suggests the transfer of genetic information, in which DNA is copied into RNA (transcription), and RNA is decoded into protein (translation). From the view of self-replication, the central dogma system has multi-rounds of flow, because DNA replication proteins (DRPs) carry out DNA replication. B: Reconstitution of the central dogma system using the PURE system. In this study, chromosomal DNA replication was reconstituted by synthesizing DRPs using the PURE system. It should be noted that the present study finally showed replication of chromosomal-type DNA by *de novo* DRP synthesis, and expression of GFP from the information encoded in the replicated DNA. This indicates reconstitution of the multi-rounds of flow of the central dogma system as described in Supplementary Figure 1A.

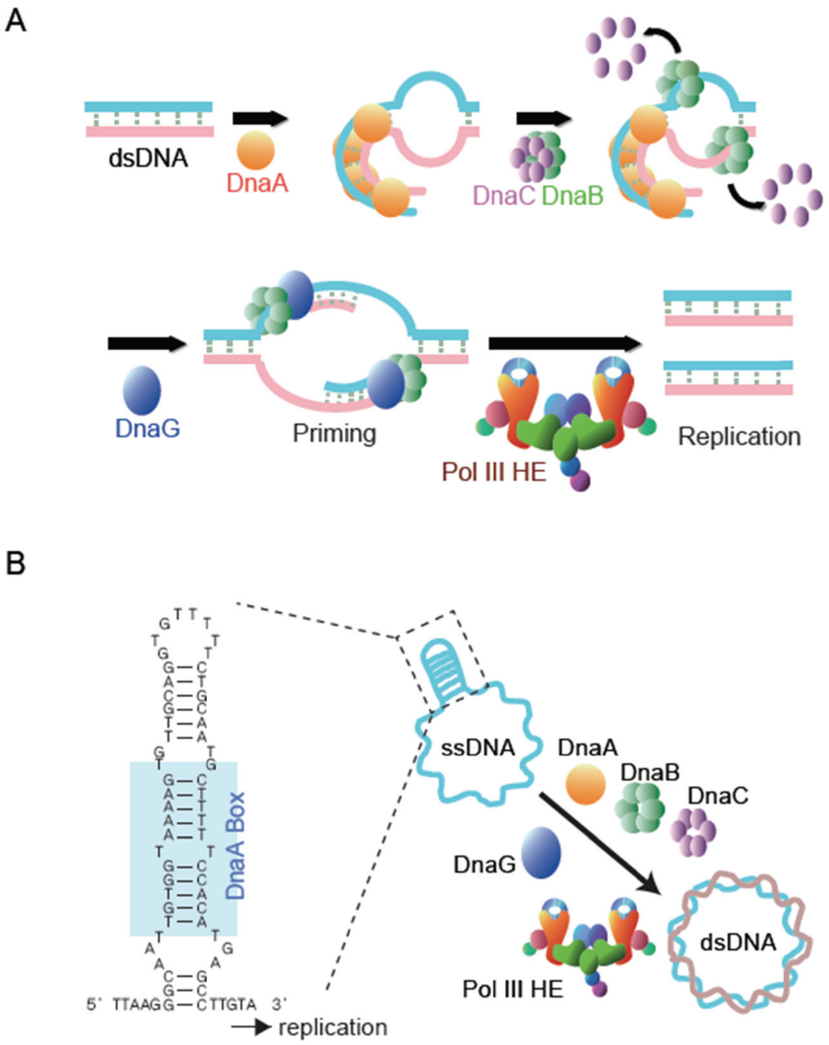

Figure S2. A: Schematic diagrams of the *E. coli* chromosomal DNA system. In *E. coli*, DnaA molecules that assemble on the origin of chromosomal replication (*oriC*) initiate chromosomal DNA replication, and then DnaC loads DnaB helicase to separate the double DNA strand. Primase (DnaG), recruited by DnaB, synthesizes RNA primers, and DNA polymerase III holoenzyme (Pol III HE) elongates the DNA chain from the RNA primer. After dissociation from DnaB, hexamers of DnaC are converted to monomers. SSB, which binds to ssDNA, is omitted from this figure for simplicity. B: Structure of A-site ssDNA. The A-site ssDNA is a single-stranded DNA with a hairpin loop that has a DnaA binding region (DnaA Box). For the replication of the A-site ssDNA, DnaA (initiator), DnaB (helicase), DnaC (helicase loader), DnaG (RNA primase), and Pol III HE complex (DNA polymerase for chromosomal DNA replication) are required. This is similar to the chromosomal DNA replication system.

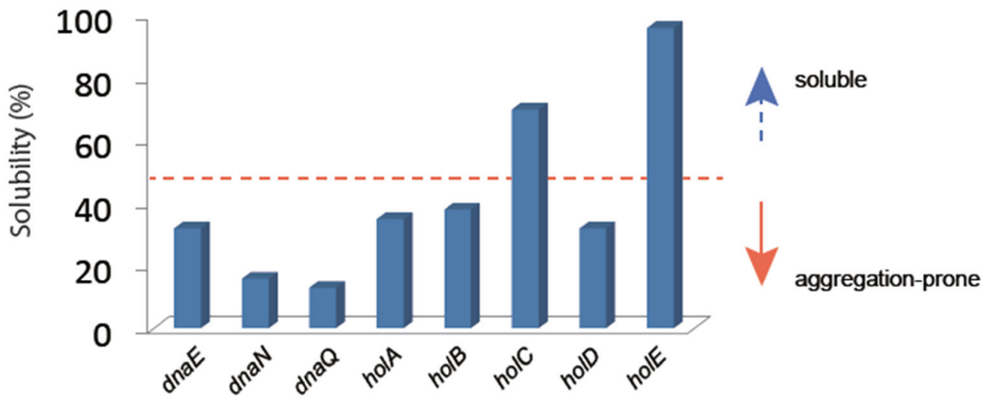

Figure S3. Proteins of Pol III HE are inherently aggregation-prone

Solubility was estimated by protein expression using the PURE system (22). The solubility of proteins contained in the Pol III HE is shown. Pol III HE consists of DnaE, DnaN, DnaX, DnaQ, HolA, HolB, HolC, HolD, and HolE. The solubility of DnaX is not shown because of too low expression yields to estimate solubility under their condition.

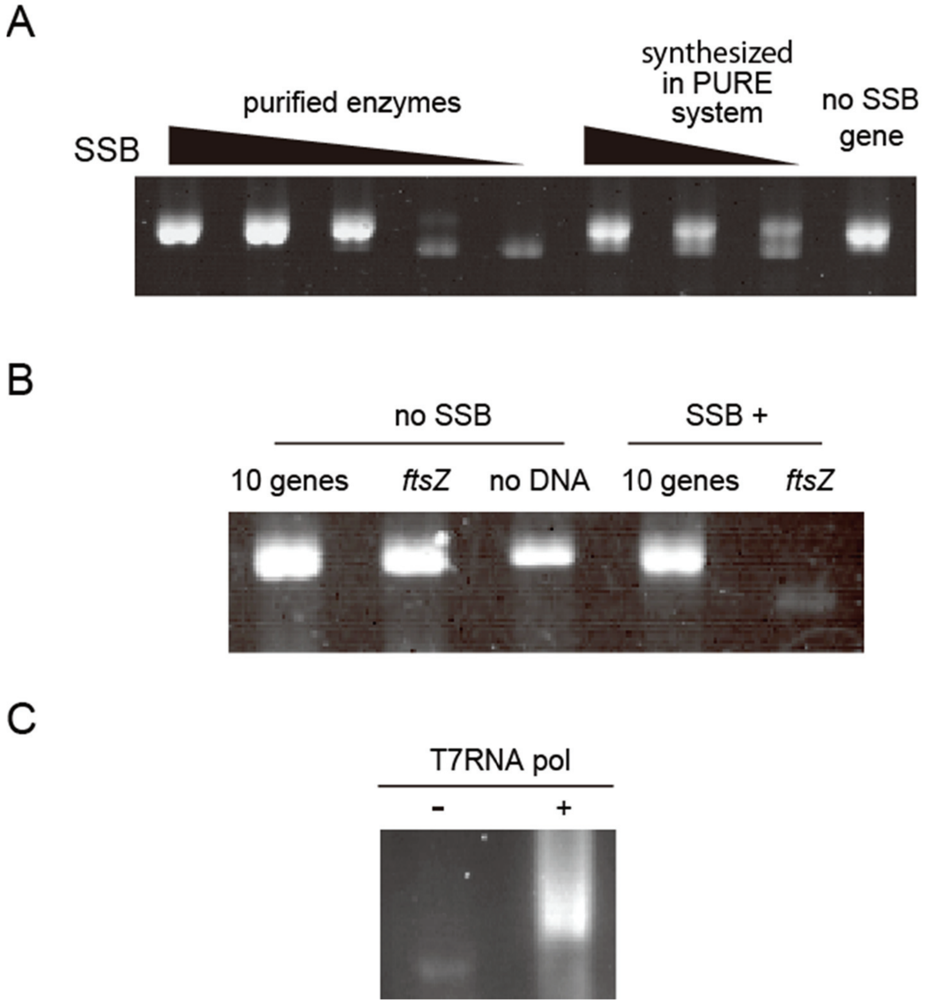

Figure S4. PURE system without SSB alters the ssDNA form

A: Replacement assay for SSB. The bands around dsDNA were formed along with the amount. B: G4 ssDNA and PURE system without dNTP were mixed, and incubated 5 min. C: Buffers of the PURE system and G4 ssDNA were mixed and incubated for 1h with (+) or without (-) T7RNA polymerase (TAKARA) at 27 °C in the absence of dNTPs. Samples were loaded on agarose-gels. A, B: stained with Ethidium bromide, C: stained with SYBR Gold.

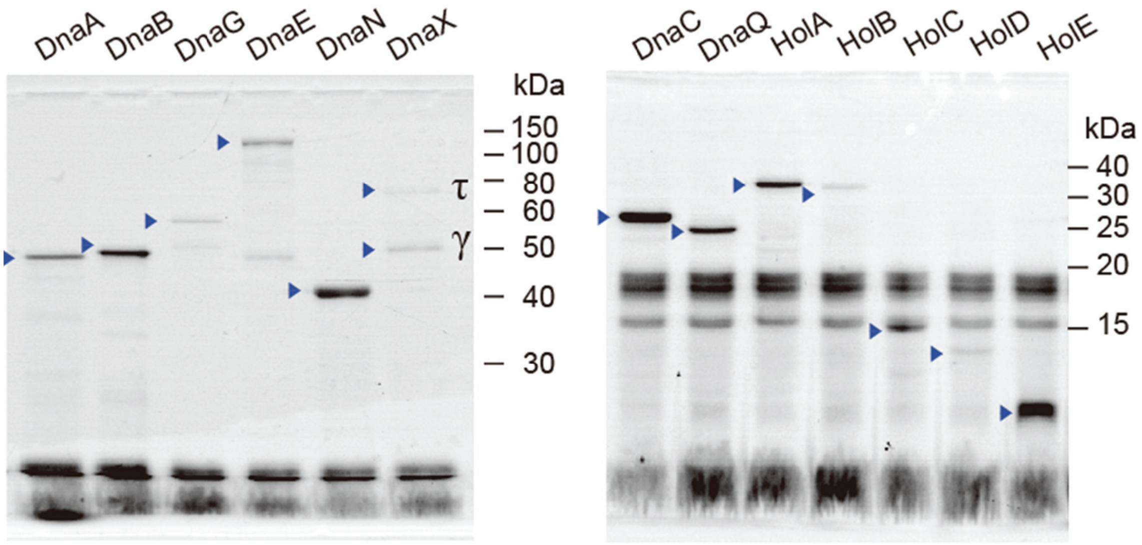

Figure S5. DNA replication proteins synthesized by PURE system

Expression of all proteins used in this study was confirmed by fluorescence tagging of synthesizing protein (FluorTect™ GreenLys in vitro Translation Labeling, Promega). In this assay, fluorescence tagged lysine were incorporated into newly synthesized proteins during translation by PURE system, followed by SDS-PAGE and fluorescence image analysis. Common bands shown from 20 to 15 kDa were fluorescence tagged lysyl tRNA used. Blue triangles indicate corresponding band of the protein. In the case of DnaX, bands that corresponded to tau (τ) and gamma (γ) subunits were observed. Smaller protein, gamma subunit of DnaX, has been shown to be expressed through spontaneous frameshift during translation (36-38).

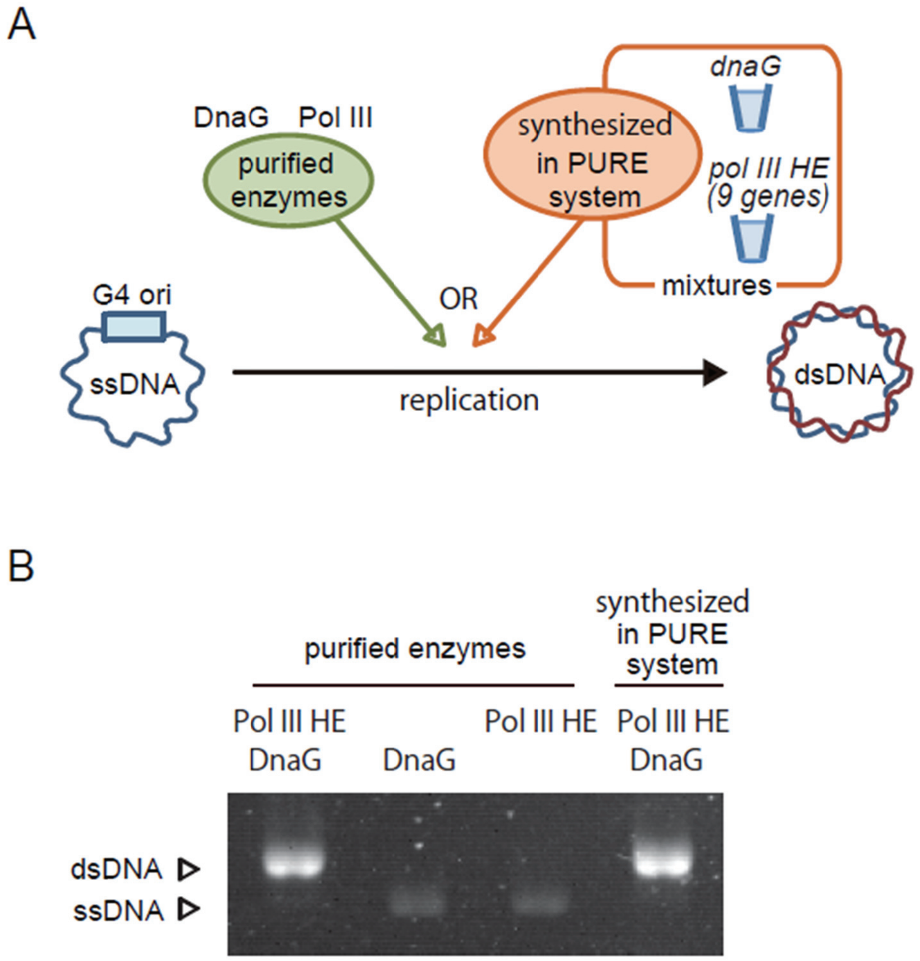

Figure S6. Mixtures of *de novo* synthesized DnaG and Pol III HE through PURE system replicates G4 DNA

A: Schematic illustration of the replication by DnaG and Pol III HE that are synthesized in PURE system. Each gene was expressed in different tubes, and then mixed. DNA replication was evaluated by G4 ssDNA. B: Replication of G4 ssDNA by DnaG and Pol III HE that are synthesized in the PURE system. After the replication reaction, DNAs were purified through SDS and $CHCl_3$/Phenol treatment. The purified DNAs were loaded into agarose-gel, were examined by electrophoresis, and were stained with Ethidium bromide.

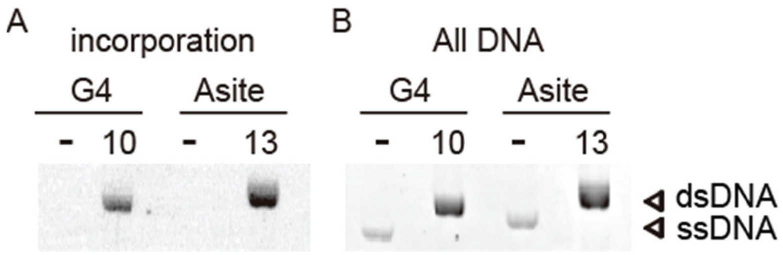

Figure S7. Incorporation of dNTP in newly synthesized DNA by DNA replication proteins synthesized in the PURE system

Incorporation of dNTP in newly synthesized DNA was confirmed by using fluorescence tagged dUTP (dUTP-ATTO488, Jena Bioscience, German). In this assay, 100 μM dTTP at final concentration was replaced with 10 μM dUTP-ATTO488 and 90 μM dTTP. G4: G4 ssDNA was used as a single strand DNA. Asite: A-site ssDNA was used as a single strand DNA. 10: 10 genes for PolIII HE and DnaG were added in the PURE system. 13: 13 genes for PolIII HE, DnaG, DnaA, DnaB, and DnaC were added in the PURE system. Minus: genes were not added. A: fluorescent image after replication without staining. Fluorecent DNA shows newly synthesized DNAs that incorporate dUTP-ATTO488. B: DNAs in the same gel as Fig. S7A were stained with MIDORI Green Gel stain (NIPPON Genetics, Japan) after taking the fluorescent image in Fig. S7A.

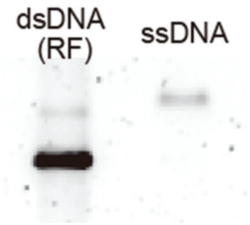

Figure S8. Single stranded DNA of T7GFP-A-site DNA migrates slower than double stranded form.

RF: replicative form of T7GFP-A-site DNA purified from phage-infected *E. coli* JM109. DNAs were stained with SYBR Gold gel stain.
